# 

**Published:** 1898-08-13

**Authors:** 


					THE HOSPITA L. ? The standard of highest purity." ?Lancet. August 13th, 1898.
y CADBURY'S ^ O ^ CADBURY'S
COOOA
ABSOLUTELY PURE.
NO DRUGS OR ALKALIES USED.1 1
COCOA
URWICH HOSPUAJ.
No, 620. Vol. XXIV. [J3SSJS] Satuedat,
August 13th, 1898.
[s
ubscription Tenng, j paICE TWOPENCE
HG6 p? oo^i J

				

